# Effects of β-Glucans Ingestion on Alveolar Bone Loss, Intestinal Morphology, Systemic Inflammatory Profile, and Pancreatic β-Cell Function in Rats with Periodontitis and Diabetes

**DOI:** 10.3390/nu9091016

**Published:** 2017-09-14

**Authors:** Viviam de O. Silva, Raquel V. Lobato, Eric F. Andrade, Débora R. Orlando, Bruno D.B. Borges, Márcio G. Zangeronimo, Raimundo V. de Sousa, Luciano J. Pereira

**Affiliations:** 1 Department of Veterinary Medicine, Federal University of Lavras (UFLA), Lavras 37200-000, Minas Gerais, Brazil; vivian_osbio@yahoo.com.br (V.d.O.S.); vieira.raquel@gmail.com (R.V.L.); ericfrancelinoandrade@gmail.com (E.F.A.); deboraribeiro.orlando@gmail.com (D.R.O.); zangeronimo@dmv.ufla.br (M.G.Z.); rvsousa@dmv.ufla.br (R.V.d.S.); 2 Department of Health Sciences, Federal University of Lavras (UFLA), Lavras 37200-000, Minas Gerais, Brazil; bruno.borges@dsa.ufla.br

**Keywords:** periodontitis, prebiotics, inflammation, bone resorption, immune system

## Abstract

This study aimed to evaluate the effects of β-glucan ingestion (*Saccharomyces cerevisiae*) on the plasmatic levels of tumor necrosis factor-α (TNF-α) and interleukin-10 (IL-10), alveolar bone loss, and pancreatic β-cell function (HOMA-BF) in diabetic rats with periodontal disease (PD). Besides, intestinal morphology was determined by the villus/crypt ratio. A total of 48 Wistar rats weighing 203 ± 18 g were used. Diabetes was induced by the intraperitoneal injection of streptozotocin (80 mg/kg) and periodontal inflammation, by ligature. The design was completely randomized in a factorial scheme 2 × 2 × 2 (diabetic or not, with or without periodontitis, and ingesting β-glucan or not). The animals received β-glucan by gavage for 28 days. Alveolar bone loss was determined by scanning electron microscopy (distance between the cementoenamel junction and alveolar bone crest) and histometric analysis (bone area between tooth roots). β-glucan reduced plasmatic levels of TNF-α in diabetic animals with PD and of IL-10 in animals with PD (*p* < 0.05). β-glucan reduced bone loss in animals with PD (*p* < 0.05). In diabetic animals, β-glucan improved β-cell function (*p* < 0.05). Diabetic animals had a higher villus/crypt ratio (*p* < 0.05). In conclusion, β-glucan ingestion reduced the systemic inflammatory profile, prevented alveolar bone loss, and improved β-cell function in diabetic animals with PD.

## 1. Introduction

Nowadays, there is an increasing interest in investigating the role of fibers and other prebiotics for preventing or controlling chronic diseases. A single active compound present in these natural products may perform several functions in the organism. Within all types of fibers, β-glucans have been used with potential effects on the immune and metabolic system.

Periodontal disease and diabetes mellitus are major public health-related chronic diseases, prevalent worldwide. Both have multifactorial etiology, and evidence indicates a bidirectional relationship between them [1,2,3]. Individuals with type 2 diabetes are 2.81 times more likely to experience the loss of clinical insertion and 3.43 times more likely to show radiographic bone loss when compared to healthy controls [4,5]. Moreover, diabetic patients with uncontrolled blood glucose levels present an increased risk of alveolar bone loss and more severe progression of periodontal disease in comparison to normoglycemic ones [6]. On the other hand, periodontal disease also affects glycemic control in diabetic patients [5,6].

Although the relationship between periodontal disease and diabetes is still unclear, it is known that patients with diabetes commonly show a change in monocyte/macrophage function, with an increased production of cytokines in response to periodontal pathogens [5,7]. These inflammatory cytokines destroy the supporting tissues of the teeth, promoting the progressive loss of attachment and bone loss, thus leading to the clinical signs of the disease [8]. Several inflammatory cytokines, such as pro-inflammatory and anti-inflammatory interleukins, are involved in periodontal diseases [9]. Interleukin-10 (IL-10) is an anti-inflammatory cytokine and a key moderator of inflammation. On the other hand, tumor necrosis factor α (TNF-α) is a pro-inflammatory cytokine, and overexpression may cause the degradation of periodontal tissue [8]. In this context, developing new ways of controlling both conditions simultaneously is of high interest.

Based on previous results by our research group [10], treatment with β-glucans has generated metabolic and inflammatory benefits in diabetic rats with periodontal disease. However, our studies have not yet evaluated the systemic effect of β-glucans on immunity, such as the determination of serum levels of pre and pro-inflammatory cytokines. Since the mediators originated by the periodontal tissue may affect insulin secretion and/or resistance, evaluating their effect on pancreatic β-cell function is mandatory [10,11].

Some studies emphasize that certain types of fibers, such as β-glucans, besides positively modulating the immune system, may act directly on the intestinal mucosa, improving the integrity of the epithelial layer, increasing the absorption of minerals, and reducing colonization by pathogenic microorganisms [12,13].

To elucidate the mechanism involved in β-glucans action, this study aimed to evaluate the immune response through a determination of the blood levels of interleukins like TNF-α and IL-10; intestinal morphology; alveolar bone loss via scanning electron microscopy and histometry; and pancreatic β-cell function in diabetic rats with periodontal disease while ingesting β-glucans from *Saccharomyces cerevisiae*.

## 2. Materials and Methods

### 2.1. Animals

Forty-eight adult healthy male rats (*Rattus norvegicus albinus*, Wistar), weighing 203 + 18 g, from the Animal Laboratory of the Federal University of Lavras, were used. A completely randomized design was employed in a 2 × 2 × 2 factorial scheme (diabetic or normoglycemic, with or without periodontal disease, treated or not with β-glucan), with six animals per treatment (*n* = 6). After acclimatization (seven days), animals were randomly divided into eight groups, according to Table 1. This study was approved by the Ethics Committee on Animal Use of the Federal University of Lavras (CEUA protocol 083/11), in accordance with the current national legislation of the National Council of Control of Animal Experimentation (CONCEA).

### 2.2. Diabetes Induction

**Figure 1:**
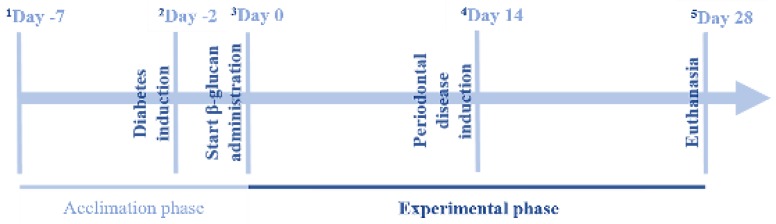
Schematic representation of the experimental design across time. ^1^ All animals were acclimated in individual metabolic cages for seven days; ^2^ Diabetes induction (80 mg/kg of streptozotocin) for animals in the following groups: diabetes, diabetes + periodontal disease, diabetes + β-glucan, diabetes + periodontal disease + β-glucan; ^3^ Start of experimental period-start of β-glucan administration for animals in the following groups: β-glucan, diabetes + β-glucan, periodontal disease + β-glucan, diabetes + periodontal disease + β-glucan; ^4^ Periodontal disease induction (by ligature protocol) for animals in the following groups: periodontal disease, diabetes + periodontal disease, periodontal disease + β-glucan, diabetes + periodontal disease + β-glucan; ^5^ Euthanasia-collection of blood and tissue samples and removal of the jaws.

Half of the animals received an administration of an intraperitoneal injection of streptozotocin (80 mg/kg, Sigma, St. Louis, MO, EUA), dissolved in citrate buffer [14], 48 h before the experiment. Animals with fasting glycemia (8 h) above 200 mg/dL [10] were considered diabetic (Figure 1).

### 2.3. Induction of Periodontal Disease

Periodontal disease was induced in the right and left hemi-mandibles of the first molar by ligature protocol. The animals were anesthetized with an intraperitoneal injection of xylazine hydrochloride associated with ketamine hydrochloride (10 and 80 mg/kg, respectively). After being anesthetized, the animals were placed on the operating table, which allowed the rats’ mouths to be kept open, facilitating access to the posterior teeth of the mandible and a cotton ligature was placed around the mandibular first molar (right and left) of each animal [10,15]. Periodontal disease was induced for 14 days (Figure 1).

### 2.4. β-Glucan

The β-glucan used came from the extract of the cell membrane of *Saccharomyces cerevisiae*: β-glucans—Minimum 60.0%; Crude Protein—Maximum 8.0%; pH (solution 2%) 4.0–7.0; Ash—Maximum 10.0/100 g. Distribution of particle size: mean—41 μm; <20 μm, 19%; 20–50 μm, 43%; 50–100 μm, 28%; 100–200 μm, 10%; >200 μm, 0%. Fluidity (seconds) was 70.2; angle of repose (degrees), 31.2; compressibility, 37%; water retention capacity (mean), 7.4; solubility rate in water, 7.9. The animals received this formulation in daily doses of 30 mg/kg by gavage for 28 days, and the group with periodontal disease received ligature during the 14th day of treatment. β-glucan was administered during the 14 days prior to induction and also during the 14 days after ligation, totaling 28 days of β-glucan treatment [16,17] (Figure 1).

### 2.5. β-Cell Function (HOMA-BF)

β-cell function (BF) was obtained by the HOMA method (Homeostasis Model Assessment) [18,19], using the following formula:

HOMA BF = (20 × fasted insulin (μU/mL))/(fasted glucose (mmoL/L) − 3.5)

At the end of the experimental period (28 days), animals were fasted for eight hours and blood glucose levels were measured by amputation of the tail tip using a glucometer (Accu-Chek^®^ Roche, Basel, Switzerland). After this, the animals were euthanized by cardiac puncture under anesthesia (50 mg/kg thiopental sodium intraperitoneally). Insulin was determined by measuring the C-peptide serum levels using an enzyme-linked immunosorbent assay (ELISA) (Millipore, Darmstadt, Germany), since Insulin and C-peptide are secreted in equimolar amounts [20].

### 2.6. TNF-α e IL-10 Measurement

Blood samples were collected for the determination of serum concentrations of TNF-α (Millipore, Darmstadt, Germany) and IL-10 (Sigma, St. Louis, MO, USA) by an enzyme-linked immunosorbent assay (ELISA). The optical density (450 nm) was obtained using a spectrophotometer (Epoch, BioTeck Instruments Inc., Winooski, VT, USA).

### 2.7. Alveolar Bone Loss by Scanning Electron Microscopy

**Figure 2:**
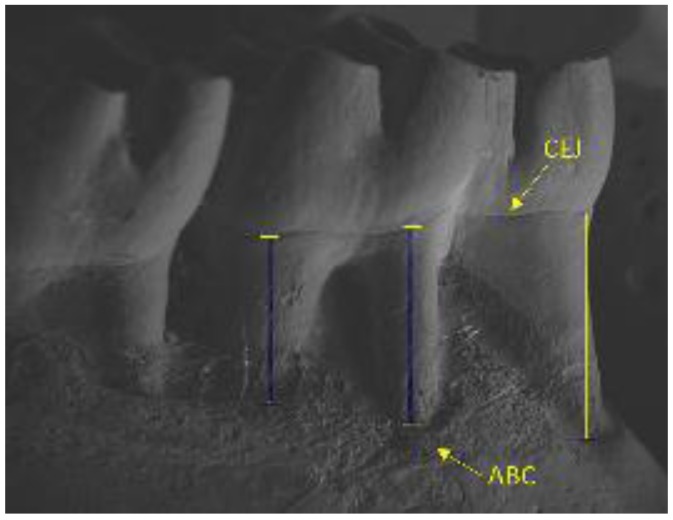
Representation of alveolar bone loss measure from the cementoenamel (CEJ) junction until the alveolar bone crest (ABC) by scanning electron microscopy.

Right mandibles were collected, dissected, and immersed in sodium hypochlorite 1% solution for four hours, and the whole remaining bone soft tissue was mechanically removed. Mandibles were inserted on stubs using double-sided carbon tape, placed on a foil wrap, and covered by a carbon bath. Images were visualized using a scanning electron microscope (LEO EVO 40, Carl Zeiss AG, Jena, Thuringia, Germany) and measurements were made using the manufacturer’s software (Smart SEM V05.09.00.00 for LEO EVO 40, Carl Zeiss AG, Jena, Thuringia, Germany). The alveolar bone loss was linearly measured from the cementoenamel junction until the alveolar bone crest (mm), at the middle point of each root, following the axis. The lingual face of the three roots of each first molar (a total of 18 roots per group) was measured and the mean of the three measurements per animal was used to express alveolar bone loss (Figure 2).

### 2.8. Histometric Analysis of Periodontal and Intestinal Tissue

Samples of tissue from the left mandible and intestine (duodenum) were collected and fixed in formalin (10%) for 48 h. Mandibles were dissected, briefly washed in water, and then placed in ethylenediaminetetraacetic acid (EDTA, 18%) demineralizing solution until complete descaling. After this, the mandibles and fractions of the intestine were processed according to histological protocol and immersed in paraffin. Serial sections of 5 μm were obtained and stained with hematoxylin and eosin for light microscopy analysis. The histological images were obtained by a digital camera connected to the microscope and analyzed using image analysis software (Image-J, Version 1.49, National Institute of Health, Bethesda, MD, USA). The area between the inter-radicular bone crest and furcation area (loss of bone mass/mm^2^) [20,21], the height of the intestinal villi, the depth of the intestinal crypt, and the villus height/crypt depth ratio [15] were measured [20].

### 2.9. Statistical Analysis

Statistical analyses were performed using Analysis of Variance (ANOVA). When *F* values indicated significant interactions, these were unfolded between factors. The analyses were performed in the statistical program SISVAR [22] with a significance level fixed at *p* < 0.05. The results were presented as the means followed by standard deviation and *n* = 6.

## 3. Results

β-cell function was lower in diabetic rats, confirming diabetes induction. In diabetic animals, β-glucan improved β-cell function. Periodontal disease induction reduced pancreatic function (*p* < 0.05; Table 2).

TNF-α and IL-10 blood levels increased because of diabetes and periodontal disease (*p* < 0.05; Table 3). Treatment with β-glucans reduced the TNF-α blood levels in diabetic animals with periodontal disease and IL-10 levels in diabetic and nondiabetic animals with periodontal disease (*p* < 0.05; Table 3).

**Figure 3:**
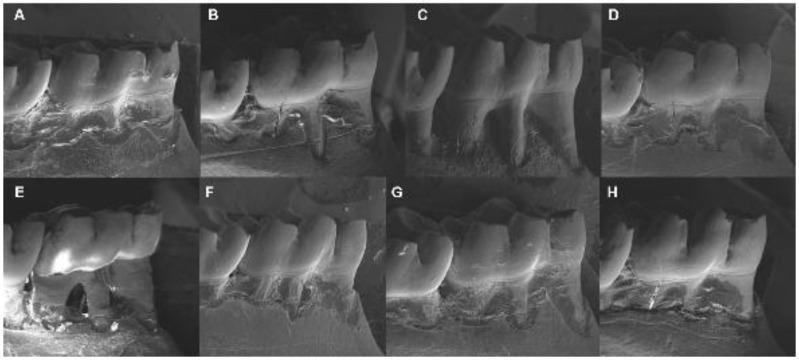
Scanning electron microscopy showing the alveolar bone loss of animals with diabetes and/or periodontal disease treated or not treated with β-glucan at a dose of 30 mg/kg/day for 28 days: (**A**) control; (**B**) diabetes; (**C**) periodontal disease; (**D**) β-glucan; (**E**) diabetes + periodontal disease; (**F**) diabetes + β-glucan; (**G**) periodontal disease + β-glucan; (**H**) diabetes + periodontal disease + β-glucan.

**Figure 4:**
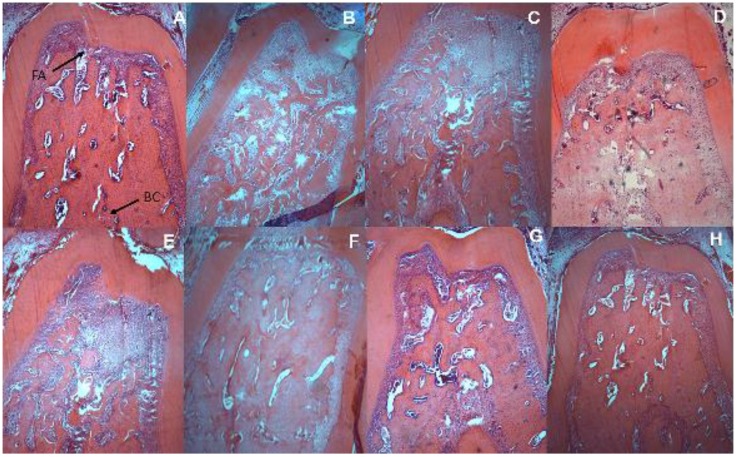
Histometric analysis showing the alveolar bone loss of animals with diabetes and/or periodontal disease treated or not treated with β-glucan at a dose of 30 mg/kg/day for 28 days: (**A**) control—FA, furcation area and BC, bone crest; (**B**) diabetes; (**C**) periodontal disease; (**D**) β-glucan; (**E**) diabetes + periodontal disease; (**F**) diabetes + β-glucan; (**G**) periodontal disease + β-glucan; (**H**) diabetes + periodontal disease + β-glucan; (Hematoxylin and Eosin; magnification 40×).

Electron microscopy images showed that treatment with β-glucan reduced alveolar bone loss in animals without diabetes (*p* < 0.05; Table 4). Moreover, animals with periodontal disease showed evident alveolar bone loss (*p* < 0.05; Table 4). The use of β-glucan reduced alveolar bone loss (*p* < 0.05; Table 4), both in the scanning electron microscopy and in the histometric analysis (Figure 3 and Figure 4, respectively).

An increase in crypt depth was found for groups with periodontal disease (*p* < 0.05; Table 5). The villus height and the villus height/crypt depth ratio were also higher in diabetic animals (*p* < 0.05; Table 5). β-glucan only increased the villus height in animals without diabetes or periodontal disease.

## 4. Discussion

This study showed that β-glucan ingestion reduced alveolar bone loss in healthy animals, in animals with periodontal disease, and in animals with periodontal disease and diabetes. Since alveolar bone loss occurs in a three-dimensional form (affects all sides of the bone surrounding the tooth), two concomitant methods were used to determine the alveolar bone loss, in order to obtain accurate results both in the vestibular and also in the bone area between tooth roots. Scanning electron microscopy was used to measure the linear distance between the cementoenamel junction and alveolar bone crest and, histometric analysis was used to measure the area between the inter-radicular bone crest and furcation area.

Authors report that the effect of β-glucans on alveolar bone loss is related to interactions with specific receptors in leukocytes located in mucosal tissues [23] and to their neutralizing effects against bacterial endotoxins [24]. Also, studies by Breivk and collaborators have shown that solutions from *Saccharomyces cerevisiae* β-1.3/1.6-glucan (10 mg/kg/day) were able to reduce bone loss in animals. These results were attributed to the potential of β-glucan to stimulate macrophage phagocytosis and T-cell differentiation [25]. Another finding that reinforces our results was the action of Polycan (β-glucan extracted from yeast Aureobasidium pullulans) in the treatment of alveolar bone loss in Sprague-Dawley rats. Polycan was given at doses of 21.25, 42.5, and 85 mg/kg/day for 10 days after the induction of periodontal disease and indicated that doses over 42.5 mg/kg/day were effective in reducing bone loss in animals with a ligature. Authors state that Polycan reduces the pro-inflammatory cytokine concentration and inhibits the production of oxidative stress [26].

The polymicrobial nature of periodontal disease can promote chronic inflammation, not only in the oral cavity, but also in other places in the human body, such as the gut. Thus, the constituents of oral bacteria are directly related to the constituents of intestinal microbiota [27,28]. Studies suggest that innate immunity receptors, such as Toll-like receptors (TLRs), can detect a wide range of signals, like the microbe-associated molecular patterns (MAMPs), to regulate and maintain intestinal homeostasis. Interactions between intestinal epithelial barriers, commensal bacteria, and mucosal immune cells provide the basis for establishing intestinal environments that promote the formation and maintenance of homeostatic mucosal balance between quiescent and active immunity. In this way, intestinal epithelial cells may play a central role in the pathogenesis of inflammation due to their interaction with the host's immune system and the intestinal microbiota [29,30].

Alterations in the intestinal barrier may be related to the systemic immune response and metabolic conditions. In this study, diabetes increased the villus height and villus height/crypt depth ratio. This ratio can be considered an indicator of the capacity of the intestinal mucosa to absorb nutrients, and thus the higher the ratio, the higher the absorption of nutrients and the lower the energy losses with cell renewal [31,32]. Authors suggest that, in diabetic individuals, the use of glucose is reduced by the tissues despite of insulin resistance or deficiency, and that the intestine tends to increase the absorption of nutrients [33,34]. We also observed a deeper crypt depth in animals with periodontal disease treated with β-glucan. Beneficial effects in the systemic immune response against pathogens by β-glucan ingestion may be related to alterations in the intestinal barrier. Since β-glucan acts in the intestine forming a layer close to the epithelium, which promotes its thickening [35,36,37], we can suppose that the deeper crypt occurred as a result of this layer formed by the ingestion of β-glucan, which may act as a barrier against pathogenic microorganisms.

Although β-glucans do not exert a direct action on intestinal morphology, these compounds can cause favorable modifications in the microbiota. Soluble fibers (currently classified as prebiotic substances) especially stimulate colonies of bacteria beneficial to our body. Because they are not digestible by humans and other species, the fibers are fermented by intestinal microorganisms, generating short-chain fatty acids (SCFAs). SCFAs produce immunoglobulin A and immunosuppressive cytokines that act systemically, playing an anti-inflammatory role [13,38,39]. The unbalance between the beneficial and pathogenic microorganisms present in the human intestine, caused by a poor fiber intake, may increase the incidence of inflammatory diseases, including diabetes. In this context, studies show that patients with type 2 diabetes present a lower number of bacteria involved in the production of SCFAs. These SCFAs are associated with a reduction in the production of glucagon-like peptide-1 (GLP-1), which in turn decreases the secretion of glucagon and improves insulin sensitivity [40,41,42]. Since the mucosal barrier functions as the primary defense mechanism of the body and is also responsible for producing immunoregulatory signals, the regular intake of fibers, such as β-glucans, can generate numerous human health benefits [13,43].

Evidence shows that chronic periodontitis is associated with mild systemic inflammation [44,45]. Therefore, measuring the serum levels of both anti and pro-inflammatory cytokines can determine the systemic inflammatory state of patients with periodontal disease, as well as to assess the systemic action of β-glucans. TNF-α and IL-10 are known to exhibit essential but contradictory functions (IL-10 can directly inhibit TNF-α production) in immune system responses to bacterial stimuli. Moreover, data indicate that both the increased expression and the deficiency of this interleukin are associated with autoimmune and inflammatory diseases, respectively [46,47,48]. Current data suggest that IL-10, which was previously described as inhibiting the synthesis of cytokines, plays an immunological-regulatory role. IL-10 is a β-cell stimulator, enhancing β-cell proliferation and differentiation. These facts suggest that IL10 can play important roles in the regulation of cellular and humoral immune responses [45]. On the other hand, TNF-α increases the destruction of connective tissue and alveolar bone by stimulating osteoclastogenesis and inhibiting osteoblasts function [49,50]. As observed in our results, β-glucan treatment decreased both TNF-α blood levels in animals with periodontal disease and diabetes and IL-10 levels in animals with periodontal disease. Thus, our results show that β-glucan was able to attenuate the inflammatory response observed in nondiabetic and diabetic animals with periodontal disease. However, β-glucan did not change the inflammatory response in the diabetic animals without periodontitis. Even with the decrease of anti-inflammatory cytokine IL-10, we can suggest that this action of β-glucan as a modulator of the immune system was beneficial, since we observed the prevention of alveolar bone loss in these groups.

Periodontal disease caused by gram-negative bacteria stimulates the production of inflammatory cytokines (via Toll-like receptor 2 and 4), promoting a progressive destruction of periodontal tissue [51,52]. Thus, our results suggest an improvement in the inflammatory profile of animals with diabetes and/or periodontal disease by β-glucan ingestion, related to the reduction of alveolar bone loss. Kim and collaborators showed a reduction in TNF-α levels in rats with ligature-induced periodontal disease treated with β-glucan (Polycan) daily, suggesting that the progression of periodontal disease may be delayed by antagonists to specific mediators of the host [27]. In individuals with periodontitis and type 2 diabetes, serum concentrations of TNF-α and IL-10 were higher in patients with diabetes and IL-10 levels were strongly correlated with the severity of periodontal disease [5].

In a study with diabetic mice, treatment with β-glucans of different molecular weights showed a hypoglycemic effect, improving insulin secretion and decreasing insulin resistance when compared to untreated animals [53]. The evaluation of these parameters, as well as the evaluation of the pancreatic β-cell function, can be very useful in studies with diabetic animals. A previous study from our group showed that β-glucan increased insulin secretion in diabetic animals with periodontal disease and reduced glucose levels in diabetic animals with or without periodontal disease [10]. In this study, we observed that β-glucan treatment improved β-cell function in diabetic animals, corroborating previous data and suggesting that β-glucan may be beneficial as a coadjutant factor in the treatment of diabetes.

The functionality of β-glucans in the modulation of the immune or metabolic response may be influenced by their origin. Thus, those from fungi (β-1.3/1.6) show higher immunomodulatory activity, while those from grains, cereals, and grasses (β-1.3/1.4) have characteristics that confer a greater metabolic potential [54]. This study suggests that β-glucans from yeast *Saccharomyces cerevisiae* have both immunomodulatory and metabolic properties, and can become a great ally to improve the symptoms in diabetic patients with periodontal disease. Moreover, there is no evidence of toxicity to the consumption of β-glucans, which makes this compound relatively safe for consumption [54]. Thus, β-glucans appear to be a viable alternative to the use of medicines.

## 5. Conclusions

Our results suggest that treatment with β-glucans from *Saccharomyces cerevisiae*, administered within 28 days, attenuated the alveolar bone loss observed by both scanning electron microscopy and histometric analysis. β-glucan was also efficient in decreasing the serum levels of TNF-α in diabetic animals with periodontal disease and IL-10 levels in animals with periodontal disease. In addition, an improvement in the pancreatic β-cell function of diabetic animals has been observed.

## Figures and Tables

**Table 1:** Distribution of experimental groups.

Experimental Groups
1—Control
2—Diabetes (Introduction of diabetes with streptozotocin)
3—Periodontal disease (Induction of periodontal disease for 14 days)
4—β-glucan (Treated since the first day of the experiment—28 days)
5—Diabetes + Periodontal disease
6—Diabetes + β-glucan
7—β-glucan + Periodontal disease
8—Diabetes + β-glucan + Periodontal disease

**Table 2:** Homeostasis model assessment (HOMA) of beta cell function (BF) (mean ± standard deviation) of animals treated with β-glucan at a dose of 30 mg/kg/day for 28 days.

Diabetes	Periodontal Disease	β-Glucan	
		−	+
−	−	776 (175) ^Bb^	736 (183) ^Bb^
−	+	352 (147) ^Ba^	500 (199) ^Ba^
+	−	30 (4) ^Ay^	50 (11) ^Ax^
+	+	14 (1) ^Ay^	51 (4) ^Ax^

^A,B^ Different letters in the columns indicate a significant difference between the groups with and without diabetes (*p* < 0.05); ^a,b^ Different letters in the columns indicate a significant difference between the groups with and without periodontal disease (*p* < 0.05); ^x,y^ Different letters in the rows indicate a significant difference between the groups with and without β-glucan (*p* < 0.05).

**Table 3:** Concentration of TNF-α and IL-10 (pg/mL; mean ± standard deviation) of animals with diabetes and/or periodontal disease treated or not treated with β-glucan at a dose of 30 mg/kg/day for 28 days.

Diabetes	Periodontal Disease	β-Glucan	
		−	+
TNF-α			
−	−	2.61 (0.95) ^Aa^	2.81 (0.39)
−	+	4.02 (0.64) ^Ab^	3.33 (0.92)
+	−	4.29 (1.57) ^Ba^	3.79 (0.90)
+	+	7.11 (1.51) ^Bbx^	3.05 (0.79) ^y^
IL-10			
−	−	24.62 (3.74) ^a^	20.69 (5.09) ^a^
−	+	35.56 (7.03) ^bx^	28.40 (4.98) ^by^
+	−	27.41 (2.70) ^a^	26.68 (3.51)
+	+	37.45 (8.76) ^bx^	29.04 (5.86) ^y^

^A,B^ Different letters in the columns indicate a significant difference between the groups with and without diabetes (*p* < 0.05); ^a,b^ Different letters in the columns indicate a significant difference between the groups with and without periodontal disease (*p* < 0.05); ^x,y^ Different letters in the rows indicate a significant difference between the groups with and without β-glucan (*p* < 0.05).

**Table 4:** Alveolar bone loss (mean ± standard deviation) by two different methods in animals with diabetes and/or periodontal disease treated or not treated with β-glucans at a dose of 30 mg/kg/day for 28 days.

Diabetes	Periodontal Disease	β-Glucan	
		−	+
Scanning Electron Microscopy (mm)			
−	−	0.67 (0.11) ^Bax^	0.51 (0.05) ^ay^
−	+	1.00 (1.17) ^bx^	0.70 (0.17) ^by^
+	−	0.51 (0.12) ^Aa^	0.56 (0.08) ^a^
+	+	1.14 (0.05) ^bx^	0.78 (0.07) ^by^
Histometric Analysis (mm^2^)			
−	−	1.36 (0.19) ^a^	1.39 (0.29)
−	+	2.50 (0.41) ^bx^	1.58 (0.61) ^y^
+	−	1.26 (0.20) ^a^	1.14 (0.01)
+	+	2.93 (0.21) ^bx^	1.25 (0.33) ^y^

^A,B^ Different letters in the columns indicate a significant difference between the groups with and without diabetes (*p* < 0.05); ^a,b^ Different letters in the columns a significant difference between the groups with and without periodontal disease (*p* < 0.05); ^x,y^ Different letters in the rows indicate a significant difference between the groups with and without β-glucan (*p* < 0.05).

**Table 5:** Histometric analysis of the duodenal fraction (mean ± standard deviation) of animals with diabetes and/or periodontal disease treated or not treated with β-glucan at a dose of 30 mg/kg/day for 28 days.

Diabetes	Periodontal Disease	β-Glucan	
		−	+
Villus height (µm)			
−	−	396 (76) ^Ax^	574 (97) ^Ay^
−	+	409 (71) ^A^	508 (215) ^A^
+	−	700 (137) ^B^	772 (88) ^B^
+	+	667 (226) ^B^	767 (107) ^B^
Crypt depth (µm)			
−	−	239 (38)	252 (13)
−	+	226 (43) ^x^	273 (19) ^y^
+	−	233 (36)	251 (10)
+	+	242 (63) ^x^	286 (34) ^y^
Villus height/crypt depth			
−	−	1.65 (0.08) ^A^	2.29 (0.46) ^A^
−	+	1.87 (0.53) ^A^	1.86 (0.77) ^A^
+	−	3.06 (0.80) ^B^	3.09 (0.45) ^B^
+	+	2.76 (0.71) ^B^	2.68 (0.22) ^B^

^A,B^ Different letters in the columns indicate a significant difference between the groups with and without diabetes (*p* < 0.05); ^x,y^ Different letters in the rows indicate a significant difference between the groups with and without β-glucan (*p* < 0.05).
